# A Sustainable and Low-Cost Route to Design NiFe_2_O_4_ Nanoparticles/Biomass-Based Carbon Fibers with Broadband Microwave Absorption

**DOI:** 10.3390/nano12224063

**Published:** 2022-11-18

**Authors:** Wanxi Li, Fang Guo, Yali Zhao, Yanyun Liu

**Affiliations:** Department of Materials Science and Engineering, Jinzhong University, Jinzhong 030619, China

**Keywords:** biomass-based carbon fibers, NiFe_2_O_4_ nanoparticles, electromagnetic parameters, microwave absorption performance

## Abstract

Carbon-based microwave-absorbing materials with a low cost, simple preparation process, and excellent microwave absorption performance have important application value. In this paper, biomass-based carbon fibers were prepared using cotton fiber, hemp fiber, and bamboo fiber as carbon sources. Then, the precise loading of NiFe_2_O_4_ nanoparticles on biomass-based carbon fibers with the loading amount in a wide range was successfully realized through a sustainable and low-cost route. The effects of the composition and structure of NiFe_2_O_4_/biomass-based carbon fibers on electromagnetic parameters and electromagnetic absorption properties were systematically studied. The results show that the impedance matching is optimized, and the microwave absorption performance is improved after loading NiFe_2_O_4_ nanoparticles on biomass-based carbon fibers. In particular, when the weight percentage of NiFe_2_O_4_ nanoparticles in NiFe_2_O_4_/carbonized cotton fibers is 42.3%, the effective bandwidth of NiFe_2_O_4_/carbonized cotton fibers can reach 6.5 GHz with a minimum reflection loss of −45.3 dB. The enhancement of microwave absorption performance is mainly attributed to the appropriate electromagnetic parameters with the *ε*’ ranging from 9.2 to 4.8, and the balance of impedance matching and electromagnetic loss. Given the simple synthesis method, low cost, high output, and excellent microwave absorption performance, the NiFe_2_O_4_/biomass-based carbon fibers have broad application prospects as an economic and broadband microwave absorbent.

## 1. Introduction

With the widespread application of electronic and electrical equipment, wireless communication systems, and radar stealth technology, the problems of electromagnetic pollution and electromagnetic interference have become increasingly serious, leading to the research on electromagnetic wave-absorbing materials receiving increasing attention [1,2,3]. Microwave-absorbing materials are being widely applied in both civil and military areas, and the requirements for the material preparation with low cost, high output, and ease to manufacture, and for the performance with thin thickness, lightweight, wide frequency band, and strong absorption are also higher and higher, which brings more challenges to researchers in material preparation and regulation [4,5,6,7]. According to the electromagnetic energy conversion principle, to achieve efficient and broadband absorption of electromagnetic waves, microwave-absorbing materials need to have two conditions: one is the electromagnetic waves can effectively enter the interior of microwave-absorbing materials, which involves the problem of impedance matching; the other is the absorbing material can effectively attenuate the electromagnetic waves entering its interior, which involves the issue of electromagnetic loss [8,9]. Impedance matching and electromagnetic loss are usually contradictory, and the controllable adjustment of electromagnetic parameters plays a decisive role in balancing impedance matching and electromagnetic loss [10,11,12].

In recent years, carbon materials have attracted extensive attention from microwave-absorbing material researchers because of their good chemical and thermal stability, excellent dielectric properties, and low density [13,14]. Optimizing the composite consisting of magnetic nanomaterial and carbon material can not only overcome the defect of the high density of magnetic materials such as ferrite, magnetic metal, magnetic metal oxide, and alloy, but also regulate electromagnetic parameters and improve electromagnetic matching, achieving better electromagnetic wave absorption than a single microwave-absorbing material. Various carbon materials, such as carbon fibers, carbon nanotubes, graphene, and metal organic-framework compounds have been widely used as carbon sources of carbon-based composite microwave-absorbing materials [15,16,17,18]. Biomass is a cheap, eco-friendly, and resource-rich renewable resource. After the carbonization of biomass, porous carbon materials with multi-stage pore size distribution can be obtained [19,20]. Recent studies have found that the porous carbon structure can not only reduce the density of materials, but also enhance impedance matching and achieve excellent microwave absorption performance [21,22]. Zhou et al. [23] prepared three-dimensional porous carbon/Fe_3_O_4_@Fe at different calcination temperatures using a towel gourd sponge as carbon source. When the calcination temperature was 600 °C, the minimum reflection loss (RL) of the composite was −49.6 dB with an effective bandwidth of 5 GHz. The synergistic effects of three-dimensional porous structure, interface polarization, dielectric loss, and magnetic loss contribute to the enhancement of microwave absorption performance. Therefore, it is a sustainable, ubiquitous, and low-cost method to obtain porous carbon materials from biomass. Because of the unique characteristics, such as hierarchical structure, periodic mode, and some individual nanostructures, it is desirable to prepare porous carbon microwave absorbent using biomass under mild conditions. Although some progress has been made in the exploration of such materials, the proportion regulation of porous carbon materials and magnetic components is the basis and key for the continuous and controllable regulation of electromagnetic parameters, which requires breakthroughs in the selection of carbon sources, the choice of preparation methods, and the control of process conditions.

Some previous results, both for conductive and magnetic inclusions, showed the influence of the shape of the additives in the electromagnetic behavior of the composites. Pinto et al. showed how additives based on magnetic metallic iron with the same crystallographic structure but with different morphologies (filaments and spherical particles) confer different electromagnetic characteristics [24]. Other works, such as Copper microwires composite and Fe-based amorphous magnetic microwires with a high aspect relationship allowed the obtention of sheets of thickness below 1 mm with reflection loss below −20 dB in the X-band [25,26]. Natural plant fiber materials have orderly optimized and stable morphology and structure. Moreover, the main component of natural plant fiber is cellulose, which is an economical and feasible raw material for the preparation of porous carbon fibers with multi-level pore size distribution. At present, the research of natural plant fibers in the field of microwave-absorbing materials has just started [27,28,29]. Ferrite nanoparticles have high permeability and good oxidation resistance. The electromagnetic parameters of carbon matrix composites can be continuously adjusted by regulating the loading amount of ferrite nanoparticles [30,31]. This feature can not only overcome the shortcomings of easy agglomeration and high density of magnetic materials, but also facilitate the study of electromagnetic loss mechanism and broaden the frequency band of microwave absorbents.

In this paper, we take the carbonized cotton fiber, carbonized hemp fiber, and carbonized bamboo fiber obtained by calcination of cotton fiber, hemp fiber, and bamboo fiber as carbon sources, and introduce different contents of NiFe_2_O_4_ nanoparticles, preparing a new carbon-based microwave-absorbing material with excellent microwave absorption performance by continuously adjusting electromagnetic parameters through composition design. The effects of the composition and structure of NiFe_2_O_4_ nanoparticles/biomass-based carbon fibers on electromagnetic parameters and microwave absorption properties were systematically investigated. The electromagnetic loss mechanism was also revealed, which provided theoretical support and experimental basis for designing cheap, lightweight, and efficient carbon-based microwave-absorbing materials.

## 2. Materials and Methods

### 2.1. Materials

Ferric chloride (FeCl_3_·6H_2_O), nickel nitrate (Ni(NO_3_)_2_·6H_2_O), and sodium hydroxide (NaOH) are analytical reagent grade, which were purchased from Shanghai Macklin Biochemical Co., Ltd. (Shanghai, China), Tianjin Bodi Chemical Co., Ltd. (Tianjin, China), and Tianjin Kaitong Chemical Reagent Co., Ltd. (Tianjin, China), respectively. The natural plant fibers were purchased from Jiangxi Zhonggan Medical Instrument Co., Ltd. (Nanchang, China). High purity N_2_ (Taiyuan Taineng Gas Co., Ltd., Taiyuan, China) was used as protective gas.

### 2.2. Preparation of NiFe_2_O_4_/Carbonized Cotton Fibers

NiFe_2_O_4_/carbonized cotton fibers were fabricated through a three-step process.

(1) An appropriate amount of cotton fibers were put into a porcelain boat, then calcinated at 700 °C for 3 h in a tubular electric furnace in a N_2_ atmosphere. The sample obtained was carbonized cotton fiber, named CCF.

(2) Amounts of 0.01 mol FeCl_3_·6H_2_O and 0.005 mol Ni(NO_3_)_2_·6H_2_O were dissolved in 30 mL water, and then 30 mL NaOH solution with the concentration of 2 mol/L was added under constant stirring. After stirring for 0.5 h, the precursor was put into a hydrothermal reactor and heated, at 200 °C, for 15 h in an electric blast drying oven. After cooling, washing, and drying at 60 °C for 12 h, NiFe_2_O_4_ nanoparticles were prepared.

(3) First, 0.3 g, 0.5 g, 1.0 g, and 1.5 g of NiFe_2_O_4_ nanoparticles were put into a beaker with 30 mL distilled water, respectively, and ultrasonic for 0.5 h in an ultrasonic cleaner to form NiFe_2_O_4_ suspension. Then, a certain quality of carbonized cotton fibers were put into the beaker and ultrasonicated for 1 h. Finally, the carbonized cotton fibers were taken out by tweezers, and dried at 60 °C for 6 h. Different contents of NiFe_2_O_4_ nanoparticles were loaded on the carbonized cotton fibers, and the obtained samples were named NiFe_2_O_4_/CCF-1, NiFe_2_O_4_/CCF-2, NiFe_2_O_4_/CCF-3, and NiFe_2_O_4_/CCF-4, respectively.

### 2.3. Preparation of NiFe_2_O_4_/Carbonized Hemp Fibers and NiFe_2_O_4_/Carbonized Bamboo Fibers

Carbonized hemp fibers and carbonized bamboo fibers were prepared by changing the cotton fibers to hemp and bamboo fibers under the same conditions. NiFe_2_O_4_/carbonized hemp fibers and NiFe_2_O_4_/carbonized bamboo fibers were designed with the same preparation method of NiFe_2_O_4_/CCF-2, which were named NiFe_2_O_4_/CHF and NiFe_2_O_4_/CBF, respectively.

### 2.4. Characterization

XRD-6100 powder X-ray diffractometer (XRD, Shimadzu, Kyoto, Japan) was used to analyze the phase composition of the samples. The morphology of the samples was characterized by a JSM-7001F scanning electron microscope (SEM, Japan Electronics Co., Ltd., Kyoto, Japan) with energy dispersive spectrometer (EDS, Bruker, Karlsruhe, Germany) and a JEM-1011 transmission electron microscope (TEM, Japan Electronics Co., Ltd., Kyoto, Japan). STA6000 synchronous thermal analyzer (PerkinElmer, Waltham, MA, USA) was used for thermogravimetric (TG) analysis, and the temperature ranged from room temperature to 700 °C, with a heating rate of 10 °C/min. The magnetic properties of the samples were studied by using a Lakeshore 7404 vibrating sample magnetometer (VSM, LakeShore Company, Columbus, OH, USA). For the microwave absorption performance test, the synthetic product was mixed with paraffin and pressed into a cylindrical compact (*Φ*_out_ = 7.0 mm, *Φ*_in_ = 3.0 mm). Then, the annular test sample in the coaxial mold was connected with an Agilent N5244A vector network analyzer (Agilent Technologies Inc., Santa Clara, CA, USA) to test the scattering parameters (abbreviated as S parameters, including S11, S21, S12, and S22). The complex permittivity (*ε_r_*) and the complex permeability (*μ_r_*) of the sample were calculated based on the S parameters in the vector network analyzer in the frequency range of 2–18 GHz. Based on the above-measured *ε_r_* and *μ_r_*, the transmission line theory was used to calculate the RL, which could be calculated by the following formulas: [32,33]

*Z_in_* = (*μ_r_*/*ε_r_*)^1/2^tanh[*j*(*2πfd*/*c*)(*μ_r_ε_r_*)^1/2^], (1)

RL(dB) = 20log|(*Z_in_* − *Z*_0_)/(*Z_in_* + *Z*_0_)|, (2)

where *Z_in_* is the input impedance of the absorbent, *Z*_0_ is the characteristic impedance of the free space, *f* is the frequency of the electromagnetic wave, *d* is the thickness of the absorbent, and *c* is the speed of light in free space.

## 3. Results

**Figure 1:**
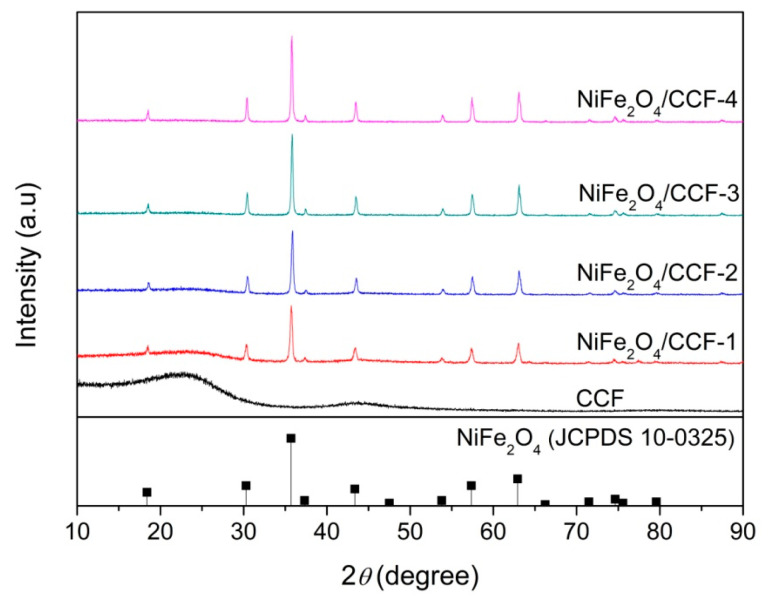
XRD patterns of CCF, NiFe_2_O_4_/CCF-1, NiFe_2_O_4_/CCF-2, NiFe_2_O_4_/CCF-3, and NiFe_2_O_4_/CCF-4.

XRD was used to analyze the phase of the samples. Figure 1 shows the XRD spectra of CCF, NiFe_2_O_4_/CCF-1, NiFe_2_O_4_/CCF-2, NiFe_2_O_4_/CCF-3, and NiFe_2_O_4_/CCF-4. For CCF, wide peaks at approximately 10°–30° and 40–50° indicate amorphous carbon [34]. For NiFe_2_O_4_/CCF-1, NiFe_2_O_4_/CCF-2, NiFe_2_O_4_/CCF-3, and NiFe_2_O_4_/CCF-4, apart from the diffraction peaks of amorphous carbon, all diffraction peaks correspond to the NiFe_2_O_4_ spinel phase (JCPDS NO.10-0325). The sharp diffraction peaks indicate the good crystallinity of NiFe_2_O_4_. From NiFe_2_O_4_/CCF-1 to NiFe_2_O_4_/CCF-4, the diffraction peaks of NiFe_2_O_4_ gradually increase, and the amorphous carbon peaks gradually weaken, indicating the increase in the loading amount of NiFe_2_O_4_. These results show that the coexistence of NiFe_2_O_4_ and amorphous carbon can be achieved by this method. In addition, the XRD patterns have no impurity peak, indicating the high purity of NiFe_2_O_4_ nanoparticles.

**Figure 2:**
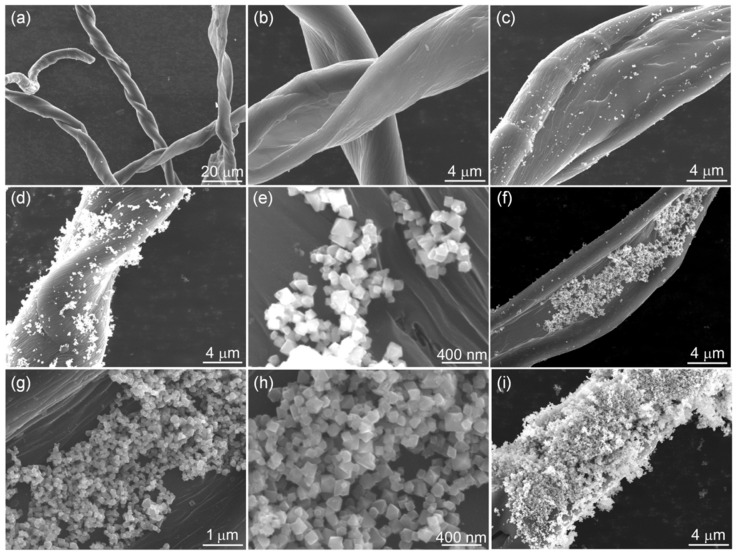
SEM images of the synthesized samples: (**a**,**b**) CCF, (**c**) NiFe_2_O_4_/CCF-1, (**d**,**e**) NiFe_2_O_4_/CCF-2, (**f**–**h**) NiFe_2_O_4_/CCF-3, and (**i**) NiFe_2_O_4_/CCF-4.

**Figure 3:**
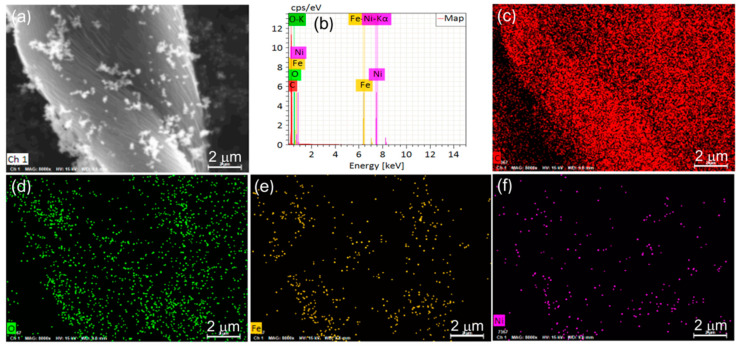
(**a**) SEM image, (**b**) EDX spectra, and (**c**–**f**) elemental mapping images of NiFe_2_O_4_/CCF-2.

The morphology of the synthetic samples was studied by SEM, as shown in Figure 2. Figure 2a,b shows the SEM images of CCF, and it is clear that the CCF is twisted and fibrous with a smooth fiber surface. Figure 2c–i shows the SEM images of NiFe_2_O_4_/CCF-1, NiFe_2_O_4_/CCF-2, NiFe_2_O_4_/CCF-3, and NiFe_2_O_4_/CCF-4. It is obvious that the NiFe_2_O_4_ nanoparticles are successfully loaded on CCF. Moreover, the NiFe_2_O_4_ nanoparticles have octahedral morphology, and the particle size is about 100 nm. From NiFe_2_O_4_/CCF-1 to NiFe_2_O_4_/CCF-4, the number of the NiFe_2_O_4_ nanoparticles on CCF shows an upward trend, indicating the increase in the loading amount of NiFe_2_O_4_ nanoparticles, which is consistent with the XRD results. The TEM images of the NiFe_2_O_4_ nanoparticles were shown in Figure S1. It is clear that the NiFe_2_O_4_ nanoparticles have clear edges and corners, and the size of the vast majority of nanoparticles is in the range of 80–120 nm. Figure 3 shows the EDS element diagram and elemental mapping images of NiFe_2_O_4_/CCF-2. It displays that the NiFe_2_O_4_/CCF-2 is mainly composed of C, O, Fe, and Ni elements, and the O, Fe, and Ni elements are displayed along the NiFe_2_O_4_ nanoparticles.

**Figure 4:**
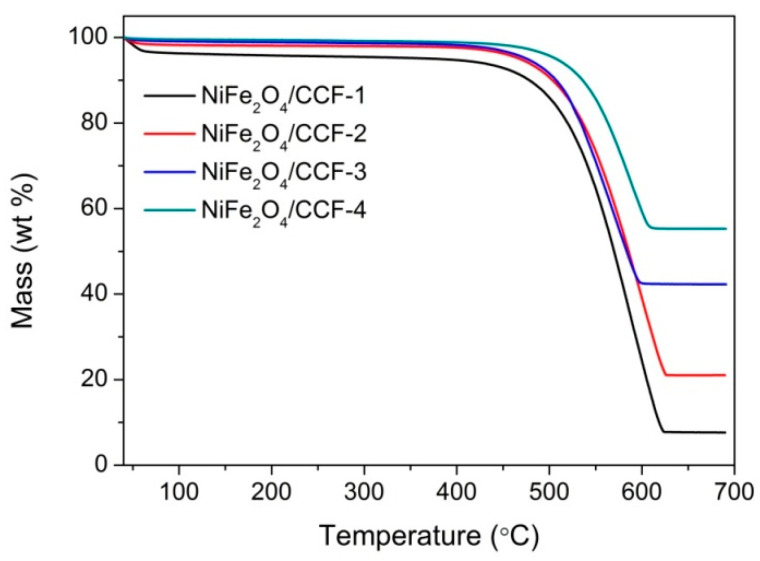
TG curves of NiFe_2_O_4_/carbonized cotton fibers in air atmosphere.

To quantitatively analyze the loading amount of NiFe_2_O_4_ nanoparticles on the NiFe_2_O_4_/carbonized cotton fibers, we studied the TG curves of NiFe_2_O_4_/carbonized cotton fibers in the air atmosphere, as shown in Figure 4. The TG curves show the mass percentage of the residue in the process of temperature change. Since the carbonized cotton fiber can be burned completely in air atmosphere, and the residual product is only NiFe_2_O_4_, the loading amount of NiFe_2_O_4_ nanoparticles can be estimated according to the TG curves. It is estimated that the loading amount of NiFe_2_O_4_ nanoparticles in NiFe_2_O_4_/CCF-1, NiFe_2_O_4_/CCF-2, NiFe_2_O_4_/CCF-3, and NiFe_2_O_4_/CCF-4 is 7.6 wt% (weight percentage), 21.0 wt%, 42.3 wt%, and 55.3 wt%, respectively. In addition, the NiFe_2_O_4_/carbonized cotton fibers have small weight loss below 450 °C, displaying good thermal stability.

**Figure 5:**
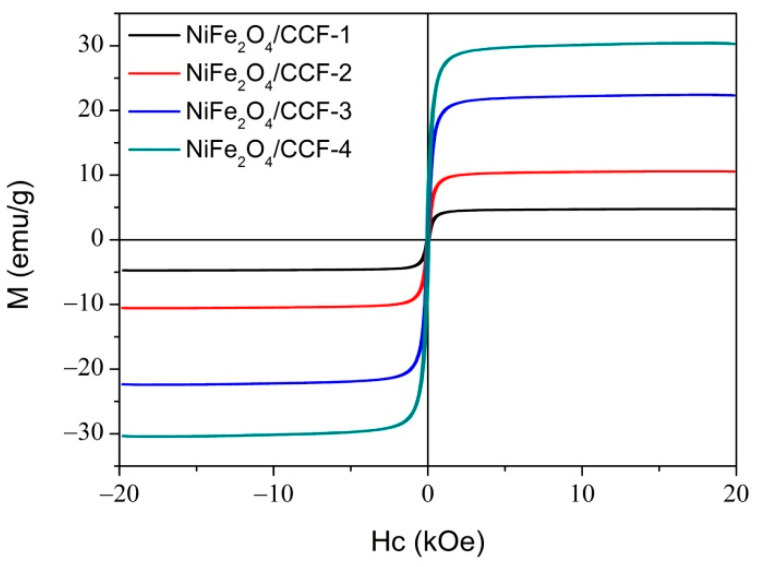
The hysteresis loops of NiFe_2_O_4_/carbonized cotton fibers.

To study the magnetic properties of NiFe_2_O_4_/CCF-1, NiFe_2_O_4_/CCF-2, NiFe_2_O_4_/CCF-3, and NiFe_2_O_4_/CCF-4, we measured the hysteresis loops at room temperature within the magnetic field of −20 kOe–20 kOe, as shown in Figure 5. It can be seen that these four samples all show ferromagnetic behavior. The saturation magnetization (*M*_S_) of NiFe_2_O_4_/CCF-1, NiFe_2_O_4_/CCF-2, NiFe_2_O_4_/CCF-3, and NiFe_2_O_4_/CCF-4 is 4.7 emu/g, 10.5 emu/g, 22.3 emu/g, and 30.3 emu/g, respectively. It is obvious that the *M*_S_ increases with the increase in the loading amount of NiFe_2_O_4_ nanoparticles, and this result is consistent with the XRD, SEM, and TG results.

**Figure 6:**
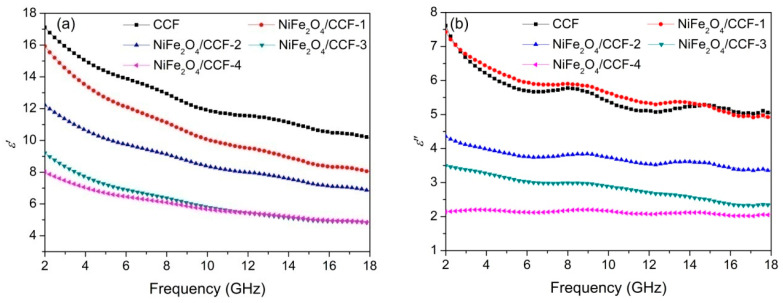

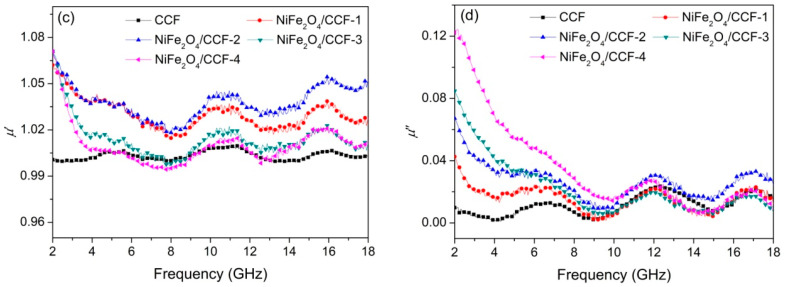
Frequency dependence of (**a**) *ε*′, (**b**) *ε*″, (**c**) *μ*′, and (**d**) *μ*″ for CCF, NiFe_2_O_4_/CCF-1, NiFe_2_O_4_/CCF-2, NiFe_2_O_4_/CCF-3, and NiFe_2_O_4_/CCF-4.

From the above analysis, NiFe_2_O_4_/carbonized cotton fibers with different loading amounts of NiFe_2_O_4_ nanoparticles were successfully prepared. Generally speaking, electromagnetic parameters are the most direct characterization parameters of the interaction between absorbing materials and electromagnetic waves, so the regulation of electromagnetic parameters is the most direct and effective means of designing absorbing materials. The most important electromagnetic parameters are complex permittivity (*ε_r_* = *ε*′ − *jε*″) and complex permeability (*μ_r_* = *μ*′ − *jμ*″). *ε*′ and *ε*″, respectively, represent the capacity and loss of electric field energy, while *μ*′ and *μ*″, respectively, represent the storage and loss of magnetic energy [35,36]. Figure 6 shows the complex permittivity and complex permeability of CCF, NiFe_2_O_4_/CCF-1, NiFe_2_O_4_/CCF-2, NiFe_2_O_4_/CCF-3, and NiFe_2_O_4_/CCF-4 with the filling ratio of 25 wt% in paraffin matrix. As can be seen from Figure 6a,b, the *ε*′ all show a downward trend in the frequency range of 2–18 GHz. This is a typical frequency dispersion phenomenon, which widely exists in carbon materials and is conducive to microwave absorption [37,38]. In addition, the *ε*′ decreases with the increase in NiFe_2_O_4_ content owing to the poor conductivity of NiFeO_4_ nanoparticles. This is because the introduction of the NiFeO_4_ nanoparticles into carbonized cotton fibers hinders the electron migration, resulting in fewer conductive network connections and reduced conductivity of the composite. For NiFe_2_O_4_/CCF-4, the *ε*″ is stable at about 2.1 in the frequency range of 2–18 GHz, while the *ε*” slowly decreases from 7.6, 7.4, 4.4, and 3.5 to 5.1, 4.9, 3.4, and 2.4, respectively, for CCF, NiFe_2_O_4_/CCF-1, NiFe_2_O_4_/CCF-2, and NiFe_2_O_4_/CCF-3. According to the Debye theory, the relationship between *ε*′ and *ε*″ can be further deduced as the following formula: [39,40].

(3) $${\left(\varepsilon^{\prime }-\frac{\varepsilon_{s}+\varepsilon_{\infty }}{2}\right)}^{2}+{\left(\varepsilon^{\prime \prime }\right)}^{2}={\left(\frac{\varepsilon_{s}-\varepsilon_{\infty }}{2}\right)}^{2}$$

where *ε*_s_ and *ε*_∞_ are static dielectric constant and dielectric constant at infinite frequency, respectively. Therefore, the Debye relaxation process can be represented by the Cole–Cole semicircle, and a semicircle represents a relaxation process. As shown in Figure S2, the CCF, NiFe_2_O_4_/CCF-1, NiFe_2_O_4_/CCF-2, and NiFe_2_O_4_/CCF-3 display two Cole–Cole semicircles, and the NiFe_2_O_4_/CCF-4 displays three Cole–Cole semicircles. The distinct Cole–Cole semicircles indicate the occurrence of the polarization relaxation process, and NiFe_2_O_4_/CCF-4 shows a stronger polarization relaxation behavior. As can be seen from Figure 6c,d, the *μ*′ and *μ*″ are very low for CCF, showing a weak storage and loss of magnetic energy in the range of 2–18 GHz. Moreover, the *μ*″ shows three vibration peaks at about 7 GHz, 12 GHz, and 17 GHz, and increases with the increase in NiFe_2_O_4_ content due to the magnetic properties of NiFe_2_O_4_ nanoparticles. Generally speaking, the magnetic loss is usually related to natural resonance, exchange resonance, and eddy current loss [41]. Eddy current loss can be determined by *C*_0_ (*C*_0_ = *μ*″*μ*′^−2^*f*^−1^ = 2π*μ*_0_*σd*^2^/3) [42,43]. If the main reason for the magnetic loss is eddy current loss, the value of *C*_0_ is constant. The *C*_0_ displays a similar vibration trend to that of *μ*″ in the frequency range 2–18 GHz (Figure S3), confirming that the natural resonance and exchange resonance are the main magnetic loss mechanism.

**Figure 7:**
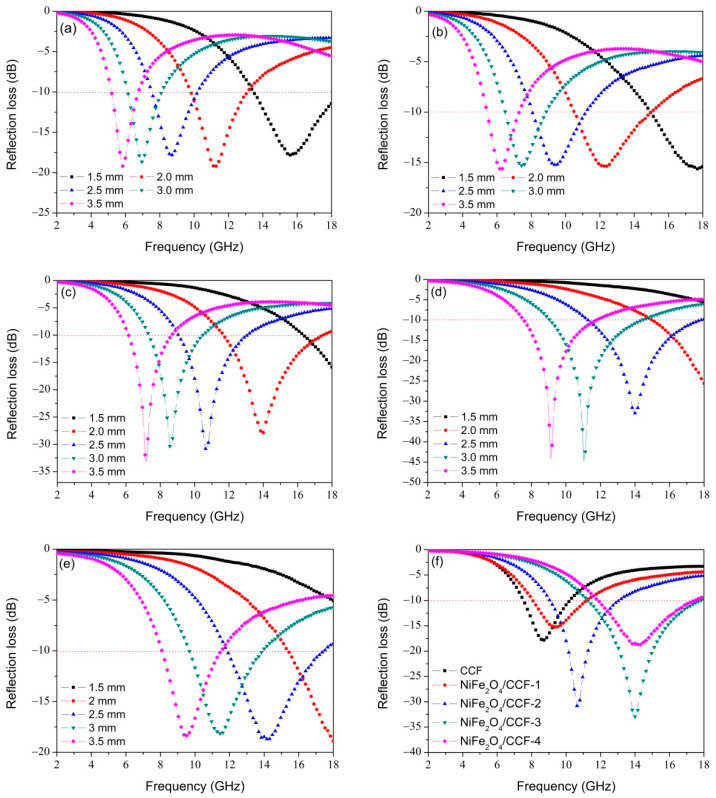
The simulated RL curves of (**a**) CCF, (**b**) NiFe_2_O_4_/CCF-1, (**c**) NiFe_2_O_4_/CCF-2, (**d**) NiFe_2_O_4_/CCF-3, and (**e**) NiFe_2_O_4_/CCF-4 at different thicknesses. (**f**) RL curves at 2.5 mm thickness for these five samples.

Generally speaking, a RL value lower than −10 dB means more than 90% microwave absorption. Only absorbent with RL lower than −10 dB can be used in practice, so the effective bandwidth represents the width of the frequency range when RL = −10 dB. According to the formulas (1) and (2) above, the RL is closely related to the thickness of the absorbent and the frequency of the electromagnetic wave. The minimum RL and effective bandwidth are obtained from the RL curve, so the minimum RL and effective bandwidth of microwave absorption are closely related to the thickness of the absorbent. Therefore, we simulated the RL curves of CCF, NiFe_2_O_4_/CCF-1, NiFe_2_O_4_/CCF-2, NiFe_2_O_4_/CCF-3, and NiFe_2_O_4_/CCF-4 at different thicknesses, as shown in Figure 7. As can be seen from Figure 7a, for CCF, the effective bandwidth is 4.5 GHz (13.5–18 GHz) at a thickness of 1.5 mm. For NiFe_2_O_4_/CCF-1, when the thickness is 2 mm, the effective bandwidth is also 4.5 GHz (10.5–15 GHz). For NiFe_2_O_4_/CCF-2, when the thickness is 2 mm, the effective bandwidth can reach 5.8 GHz (11.7–17.5 GHz). For NiFe_2_O_4_/CCF-3, when the thickness is 2.5 mm, the effective bandwidth reaches 6.5 GHz (11.3–17.8 GHz) with a minimum RL of −33.2 dB; when the thickness rises to 3 mm, the minimum RL is −45.3 dB. For NiFe_2_O_4_/CCF-4, the minimum RL is −18.8 dB with an effective bandwidth of 5.6 GHz (11.9–17.5 GHz). In addition, as shown in Figure 7a–e, it can be seen that the RL peak moves to the low-frequency region with increasing thickness. Figure 7f shows the RL curves at 2.5 mm for these five samples. Obviously, the RL peak shifts to the high-frequency region by decreasing the complex permittivity under the same thickness by comparing these five samples, and this can be explained by the geometric effect [44,45]. When the thickness (*t*_m_) of the absorbent follows the 1/4 wavelength model: *t*_m_ = *nλ*_0_/4(|*μ_r_*||*ε_r_*|)^1/2^ = *nc*/4*f*_m_(|*μ_r_*||*ε_r_*|)^1/2^(*n* =1,3,5, …), the input impedance is the same as the air wave impedance, and the RL (dB) reaches a minimum value. Simulation and calculation of the absorber thickness (*t*_m_) versus peak frequency (*f*_m_) for these five samples are shown in Figure S4, and it is obvious that the calculated results agree well with the simulated values. For the 1/4 wavelength model, the *f*_m_ is inversely proportional to the *t*_m_ and complex permittivity. For the same sample with the same *μ_r_* and *ε_r_*, the *f*_m_ is inversely proportional to the *t*_m_, so the RL curve moves to the low frequency with increasing the thickness. For different sample in the same thickness, the RL curve moves to the high frequency with decreasing the electromagnetic parameters. Thus, from CCF to NiFe_2_O_4_/CCF-4, it is understandable that the optimal RL moves to the high thickness with decreasing the complex permittivity for these five samples. As an essential standard for evaluating microwave absorption performance, the minimum RL value and effective bandwidth are also compared with other reported carbon-based absorbents, as shown in Table 1 [46,47,48,49,50,51,52,53]. Obviously, the NiFe_2_O_4_/carbonized cotton fibers show wider effective bandwidth and stronger RL with a low filling rate. What is more, compared with the microwave absorbents such as ferrite, magnetic metal, magnetic metal oxide, and alloy, the NiFe_2_O_4_/CCF has a lower density; compared with the preparation of carbon fiber, the CCF has a very simple preparation method [54]. In a word, the use of cotton fibers, simple preparation methods, and excellent microwave absorption performance give NiFe_2_O_4_/CCF broad application prospects as an economical, lightweight, and broadband microwave absorbent.

**Figure 8:**
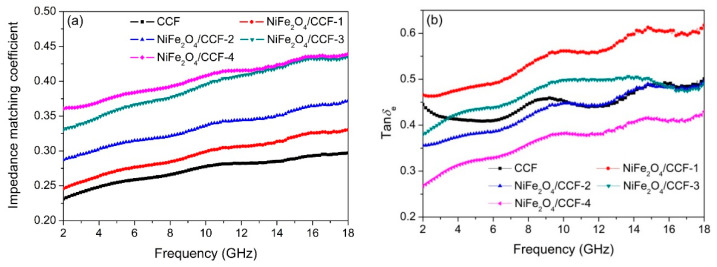

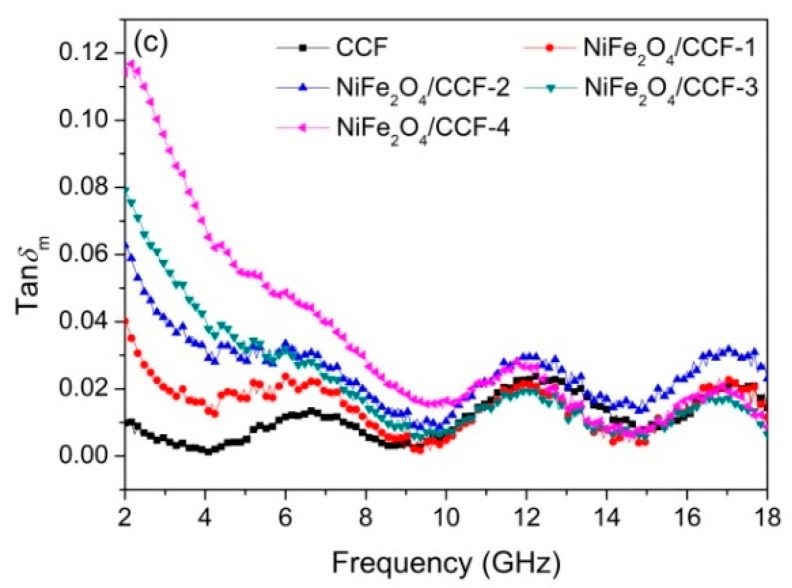
Frequency dependence of (**a**) impedance matching coefficient, (**b**) tan*δ*_e_, and (**c**) tan*δ*_m_ of CCF, NiFe_2_O_4_/CCF-1, NiFe_2_O_4_/CCF-2, NiFe_2_O_4_/CCF-3, and NiFe_2_O_4_/CCF-4.

Impedance matching and electromagnetic loss are two key factors affecting microwave absorption performance. The impedance matching can be expressed by the impedance matching coefficient (*Z* = *Z*_in_/*Z*_0_ = (*μ_r_*/*ε_r_*)^1/2^) [55,56]. The closer the *Z* is to 1, the better the impedance matching is. Recent studies have found that the *ε_r_* value is much higher than the *μ_r_* value for carbon materials [57,58]. Therefore, the decrease in the *ε_r_* can increase the impedance matching coefficient and improve the impedance matching of the absorbent. Moreover, the electromagnetic loss can be determined by the dielectric loss factor (tan*δ*_e_ = *ε*″/*ε*′) and magnetic loss factor (tan*δ*_m_ = *μ*″/*μ*′) [59,60]. Figure 8 shows the impedance matching coefficient, tan*δ*_e_, and tan*δ*_m_ of CCF, NiFe_2_O_4_/CCF-1, NiFe_2_O_4_/CCF-2, NiFe_2_O_4_/CCF-3, and NiFe_2_O_4_/CCF-4. As seen in Figure 8a, the *Z* follows the order of CCF < NiFe_2_O_4_/CCF-1 < NiFe_2_O_4_/CCF-2 < NiFe_2_O_4_/CCF-3 < NiFe_2_O_4_/CCF-4, which is opposite to the complex permittivity. So the existence of NiFe_2_O_4_ with relatively lower conductivity improves the percolation threshold in the NiFe_2_O_4_/biomass-based carbon fibers system and hence maintains a good impedance matching [61]. As seen in Figure 8b,c, it is obvious that the tan*δ*_e_ is much larger than the tan*δ*_m_, showing that the dielectric loss plays a major role. What is more, the tan*δ*_e_ shows an upward trend in the frequency of 2–18 GHz, which is highly beneficial to high-frequency microwave absorption. The tan*δ*_m_ shows a similar tendency to that of *μ*″, showing that the magnetic loss is mainly influenced by the imaginary part of the complex permeability. For CCF and NiFe_2_O_4_/CCF-1, although the tan*δ*_e_ is higher than 0.4 in the frequency of 2–18 GHz, the impedance matching coefficient is much lower than that of NiFe_2_O_4_/CCF-3, so the microwave absorption performance is slightly worse. For NiFe_2_O_4_/CCF-4, although the impedance matching is the best, the electromagnetic loss is relatively small, which is not conducive to microwave absorption. For NiFe_2_O_4_/CCF-3, the tan*δ*_e_ is only lower than NiFe_2_O_4_/CCF-1, while the tan*δ*_m_ is only lower than NiFe_2_O_4_/CCF-4, so the NiFe_2_O_4_/CCF-3 has relatively high electromagnetic loss. In addition, the low value of −45.3 dB at 11 GHz may be attributed to the high tan*δ*_e_ and tan*δ*_m_ value in the range of 10–14 GHz. Therefore, the excellent microwave absorption performance of NiFe_2_O_4_/CCF-3 can be attributed to the appropriate loading amount of NiFe_2_O_4_ nanoparticles with moderate electromagnetic parameters, and the balance of impedance matching and electromagnetic loss.

**Figure 9:**
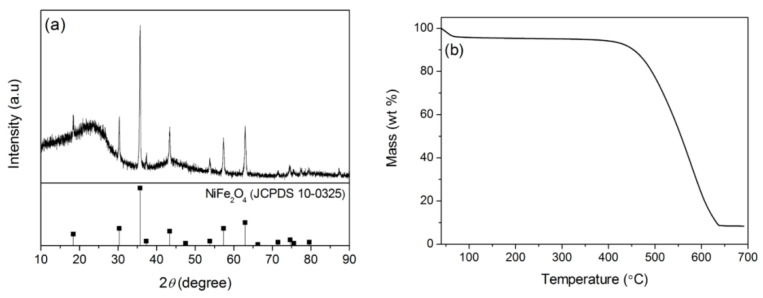
XRD pattern (**a**) and TG curve (**b**) of NiFe_2_O_4_/carbonized hemp fibers.

**Figure 10:**
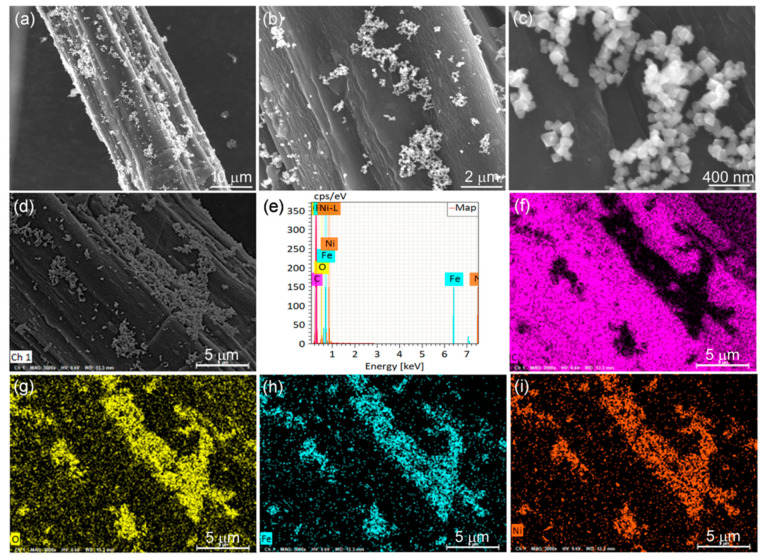
(**a**–**d**) SEM images, (**e**) EDX spectra, and (**f**–**i**) elemental mapping images of NiFe_2_O_4_/carbonized hemp fibers.

During the research, NiFe_2_O_4_/carbonized hemp fibers were also successfully prepared by changing the cotton fibers to hemp fibers under the same conditions. Figure 9a shows the XRD spectrum of NiFe_2_O_4_/CHF. In addition to the diffraction peaks of NiFe_2_O_4_, the wide peaks at 10°–30° and 40–50° are the diffraction peaks of carbonized hemp fibers. Figure 9b shows the TG curves of NiFe_2_O_4_/CHF in the air atmosphere, revealing that the loading amount of NiFe_2_O_4_ nanoparticles in NiFe_2_O_4_/CHF is 8.3 wt%. Figure 10a–d show the SEM images of NiFe_2_O_4_/CHF at different magnifications. From the SEM images, we can observe that the NiFe_2_O_4_ nanoparticles are attached to the surface of the carbonized hemp fibers. Figure 10e–i show the element diagram and elemental mapping images of NiFe_2_O_4_/CHF in Figure 10d. It displays that the NiFe_2_O_4_/CHF is mainly composed of C, O, Fe, and Ni elements, and the Fe, Ni, and O elements are distributed along the NiFe_2_O_4_ nanoparticles on the carbonized hemp fibers. In addition, when the hemp fibers were changed to bamboo fibers under the same conditions, NiFe_2_O_4_ nanoparticles were also loaded on the carbonized bamboo fibers. Moreover, the microwave absorption performance of the NiFe_2_O_4_/carbonized bamboo fibers was also investigated (See Supporting Information Figure S5–S7). In general, the experimental results indicate that this facile self-assembly technology is universally applicable in designing other types of carbon matrix composites.

**Figure 11:**
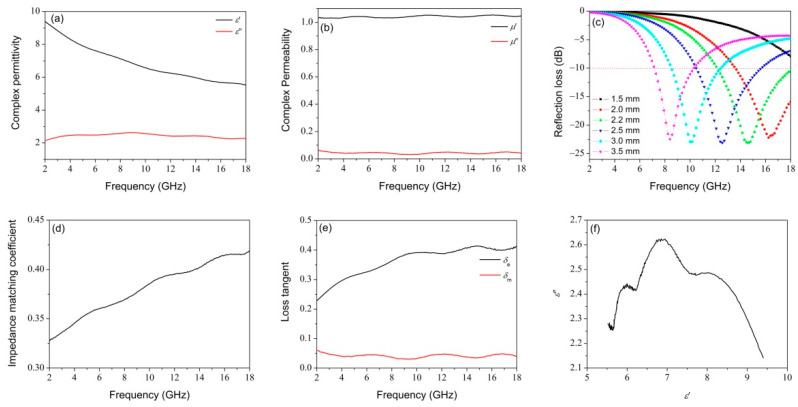
The frequency dependence of (**a**) complex permittivity, (**b**) complex permeability, (**c**) simulated reflection loss, (**d**) impedance matching coefficient, (**e**) loss factor, and (**f**) Cole–Cole curve of NiFe_2_O_4_/carbonized hemp fibers.

The electromagnetic properties of NiFe_2_O_4_/CHF dispersed in paraffin matrix with 33 wt % are shown in Figure 11. Figure 11a illustrates the complex permittivity of NiFe_2_O_4_/CHF. The *ε*′ decreases from 9.4 to 5.5, and the *ε*″ is in the range of 2.1–2.6 with three vibration peaks. Figure 11b shows the complex permeability of NiFe_2_O_4_/CHF. Interestingly, the *μ*″ shows an opposite trend to *ε*″, showing that there is an obvious electromagnetic synergistic effect, which is conducive to electromagnetic loss. Figure 11c displays the simulated RL curve of NiFe_2_O_4_/CHF at different thicknesses. The effective bandwidth is 5.85 GHz (12.15–18 GHz) with a minimum RL of −23.1 dB at a thickness of 2.0 mm. Figure 11d shows the impedance matching coefficient of NiFe_2_O_4_/CHF, which shows an upward trend in the frequency range 2–18 GHz, implying that the well-matched characteristic impedance contributes to high-frequency microwave absorption. Figure 11e illustrates the dielectric loss factor and magnetic loss factor. The curves of dielectric loss tangent and magnetic loss tangent are similar to *ε*″ and *μ*″, respectively. This illustrates that the loss tangent is mainly affected by the imaginary part of the complex permittivity and permeability. Moreover, the dielectric and magnetic loss tangent present opposite vibration peaks, indicating a strong electromagnetic coupling effect [62]. In addition, the dielectric loss tangent gradually increases, which is in favor of high-frequency electromagnetic loss. Figure 11f corresponds to the Cole–Cole curve of NiFe_2_O_4_/CHF, which has three Cole–Cole semicircles, meaning that it has multiple relaxation processes and promoted interfacial polarization. Such polarization processes are conducive to dielectric loss. In a word, the impedance matching, and the synergy between dielectric loss and magnetic loss contribute to the enhanced microwave absorption performance of NiFe_2_O_4_/CHF.

## 4. Discussion

To sum up, taking cotton fiber, hemp fiber, and bamboo fiber as the carbon source, the accurate loading of NiFe_2_O_4_ nanoparticles with different contents on biomass-based carbon fibers was successfully achieved. The results show that the impedance matching is optimized, and the microwave absorption performance is improved after loading NiFe_2_O_4_ nanoparticles on the biomass-based carbon fibers. In particular, when NiFe_2_O_4_ nanoparticles are loaded with 42.3 wt%, the effective bandwidth of the NiFe_2_O_4_/carbon cotton fiber can reach 6.5 GHz with a minimum RL of −45.3 dB. The enhancement of microwave absorption performance is attributed to the appropriate loading amount of NiFe_2_O_4_ nanoparticles with moderate electromagnetic parameters, and the balance of impedance matching and electromagnetic loss. This study is expected to provide a new way for designing new carbon-based microwave-absorbing materials.

## Figures and Tables

**Table 1:** Microwave absorption performance of some reported carbon-based absorbents.

Sample	Filling Rate (wt%)	Effective Absorption Bandwidth (GHz)	Minimum RL (dB)	References
MOF-Derived Porous Co/C Nanocomposites	60	5.8	−35.3	[46]
NiFe_2_O_4_ hollow particle/graphene	15	4.5	−40.9	[47]
Ferrite/Co/porous carbon	70	4.8	−31.0	[48]
Fe@porouscarbon@carbonfiber	25	5.2	−46.2	[49]
RGO-PANI-NiFe_2_O_4_	30	5.3	−49.7	[50]
Ni/Carbon nanocomposites	25	4.4	−21.2	[51]
CoNi@Ccomposites	50	5.0	−35.8	[52]
CoFe_2_O_4_@graphene composites	45	4.6	−42	[53]
NiFe_2_O_4_/carbonized cotton fibers	25	6.5	−45.3	This work

## Data Availability

Not applicable.

## Supplementary Materials

The following supporting information can be downloaded at: https://www.mdpi.com/article/10.3390/nano12224063/s1, Figure S1: (a,b) TEM images of the NiFe_2_O_4_ nanoparticles in different magnification; Figure S2: Cole–Cole curves of (a) CCF, (b) NiFe_2_O_4_/CCF-1, (c) NiFe_2_O_4_/CCF-2, (d) NiFe_2_O_4_/CCF-3, and (e) NiFe_2_O_4_/CCF-4; Figure S3: Values of *C*_0_ (*C*_0_ = *μ*″*μ*′^−2^*f*^−1^) of CCF, NiFe_2_O_4_/CCF-1, NiFe_2_O_4_/CCF-2, NiFe_2_O_4_/CCF-3, and NiFe_2_O_4_/CCF-4; Figure S4: Simulation and calculation of the absorber thickness (*t*_m_) versus peak frequency (*f*_m_): (a) CCF, (b) NiFe_2_O_4_/CCF-1, (c) NiFe_2_O_4_/CCF-2, (d) NiFe_2_O_4_/CCF-3, and (e) NiFe_2_O_4_/CCF-4; Figure S5: XRD pattern of NiFe_2_O_4_/carbonized bamboo fibers; Figure S6: (a–d) SEM images, (e) EDX spectra, and (f–i) elemental mapping images of NiFe_2_O_4_/carbonized bamboo fibers; Figure S7: Frequency dependence of the simulated reflection loss for NiFe_2_O_4_/carbonized bamboo fibers at different thicknesses.
