# Supplementary material for: A Sustainable and Low-Cost Route to Design NiFe2O4 Nanoparticles/Biomass-Based Carbon Fibers with Broadband Microwave Absorption
Source: Nanomaterials (Basel). 2022 Nov 18;12(22):4063. doi: 10.3390/nano12224063 (PMC9693991; doi:10.3390/nano12224063)
Supplement: Supplementary file 1 [file nanomaterials-12-04063-s001.zip › nanomaterials-2003562-supplementary.pdf]

Supplementary Materials

Article

A Sustainable and Low-Cost Route to Design $NiFe_2O_4$ Nanoparticles/Biomass-Based Carbon Fibers with Broadband Microwave Absorption

Wanxi Li *, Fang Guo, Yali Zhao and Yanyun Liu

Department of Materials Science and Engineering, Jinzhong University, Jinzhong 030619, China
* Correspondence: liwanxi1986@163.com

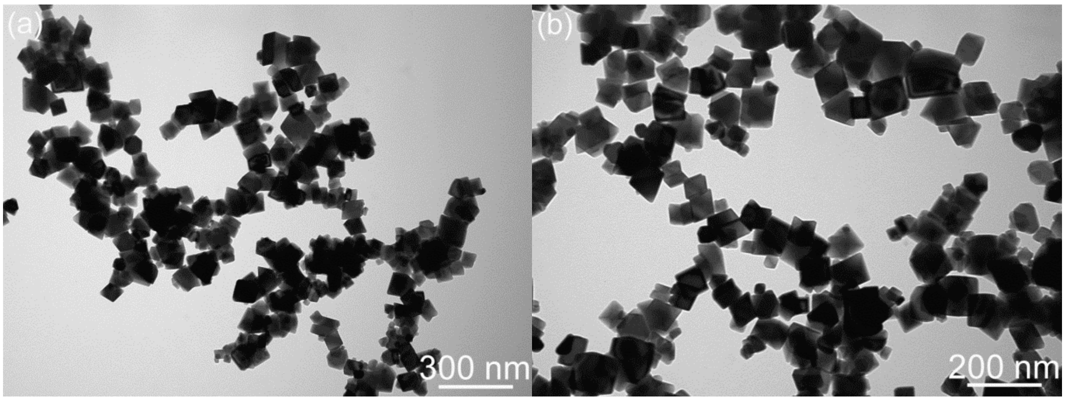

Figure S1. (**a**,**b**) TEM images of the $NiFe_2O_4$ nanoparticles in different magnification.

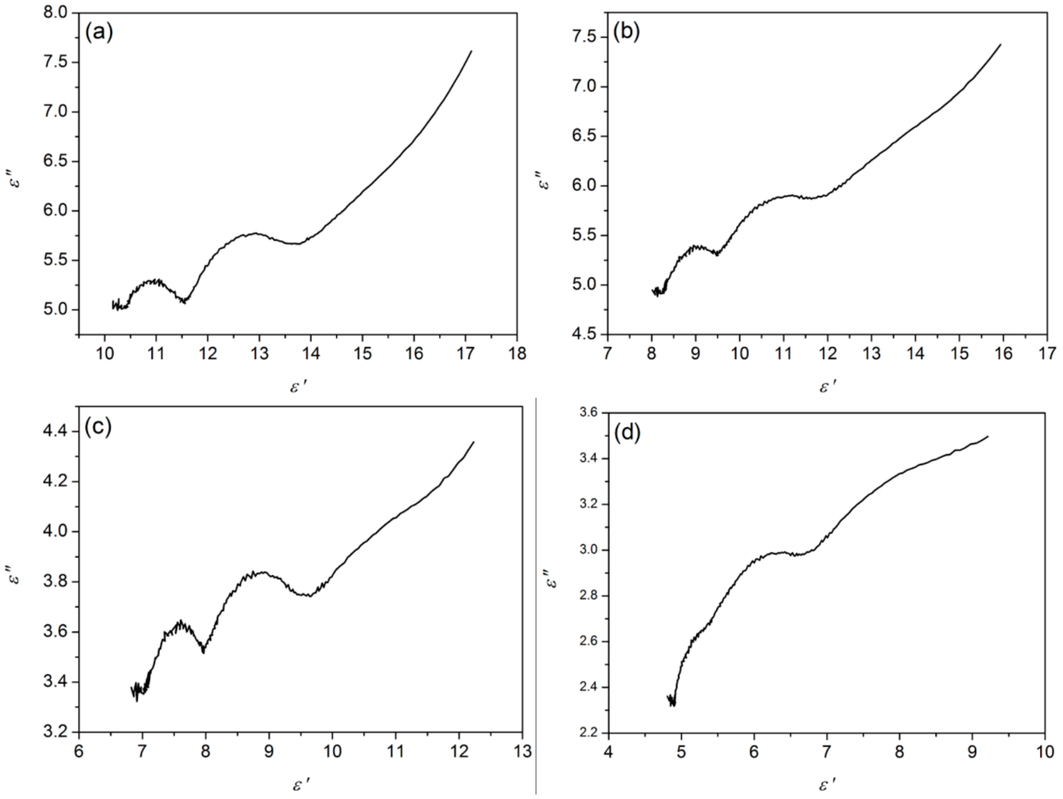

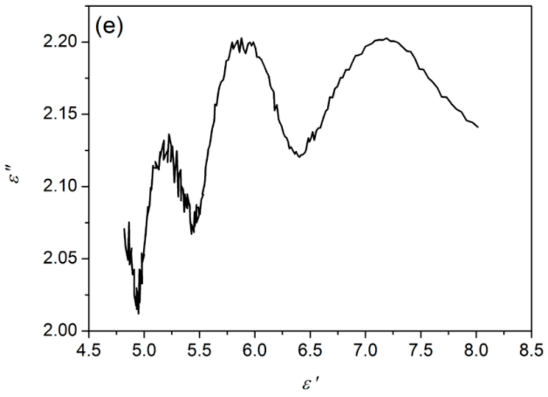

Figure S2. Cole-Cole curves of (**a**) CCF, (**b**) $NiFe_2O_4$/CCF-1, (**c**) $NiFe_2O_4$/CCF-2, (**d**) $NiFe_2O_4$/CCF-3, and (**e**) $NiFe_2O_4$/CCF-4.

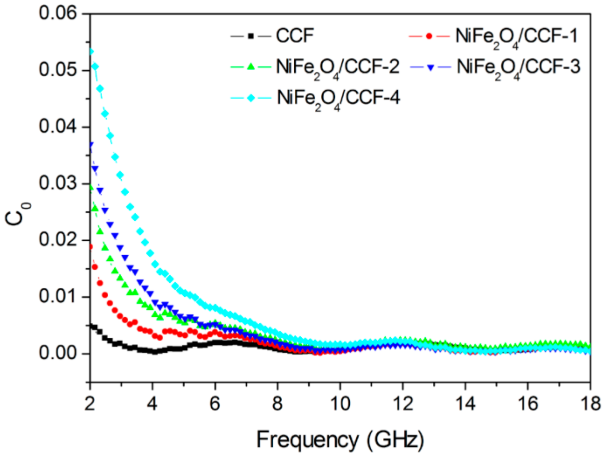

Figure S3 Values of C_0 ($C_0=\mu''\mu'^{-2}f^{-1}$) of CCF, $NiFe_2O_4$/CCF-1, $NiFe_2O_4$/CCF-2, $NiFe_2O_4$/CCF-3, and $NiFe_2O_4$/CCF-4.

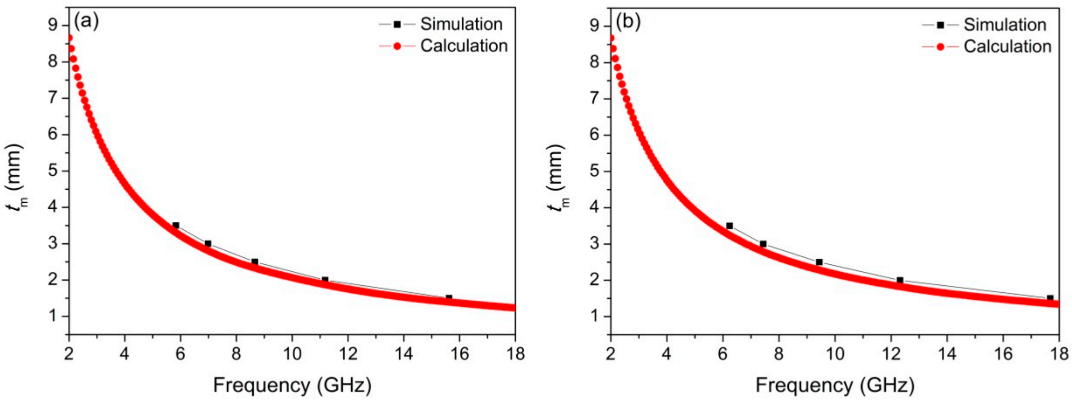

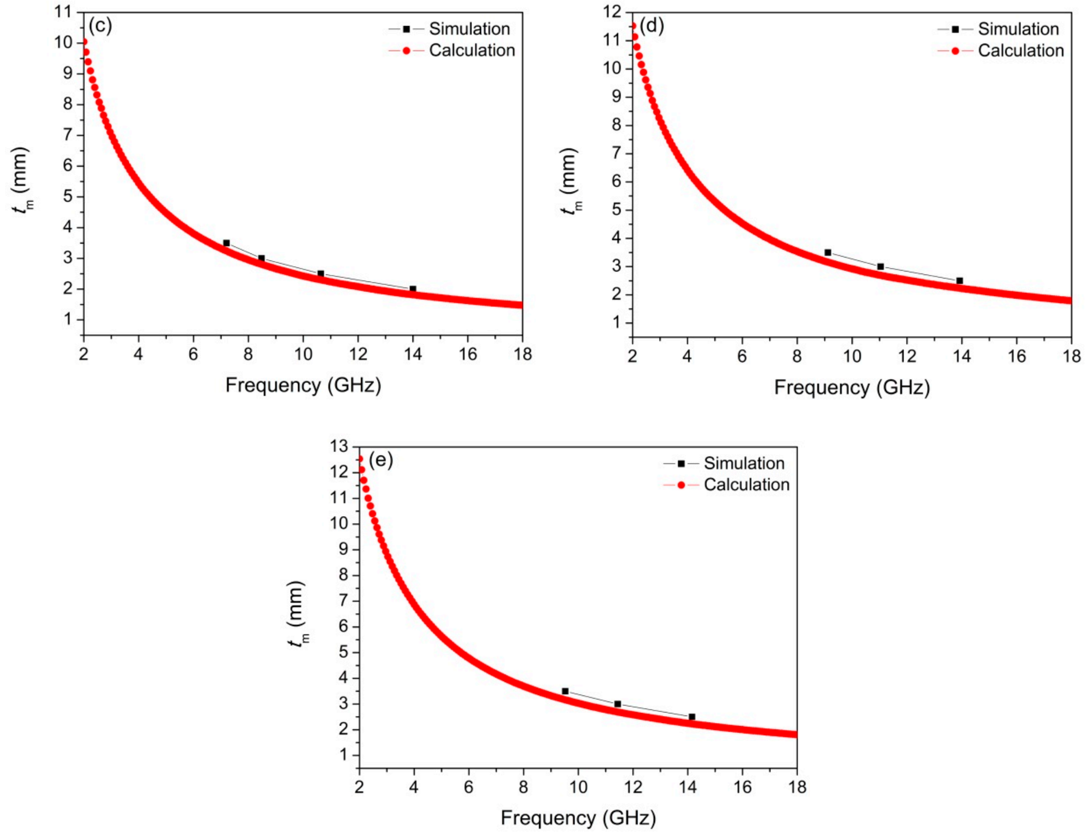

Figure S4. Simulation and calculation of the absorber thickness (t_m) versus peak frequency (f_m): (**a**) CCF, (**b**) $NiFe_2O_4$/CCF-1, (**c**) $NiFe_2O_4$/CCF-2, (**d**) $NiFe_2O_4$/CCF-3, and (**e**) $NiFe_2O_4$/CCF-4.

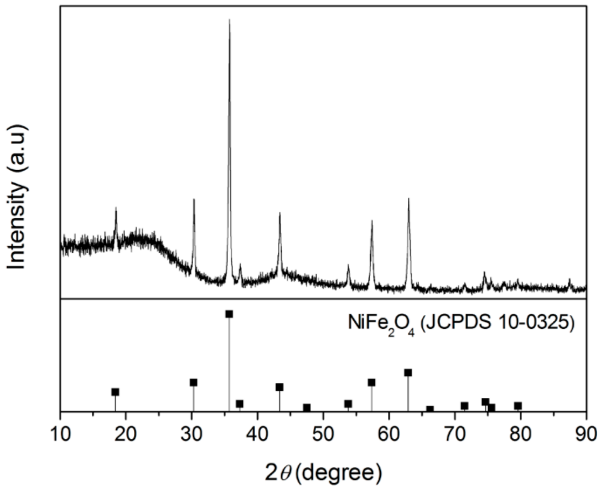

Figure S5. XRD pattern of $NiFe_2O_4$/carbonized bamboo fibers.

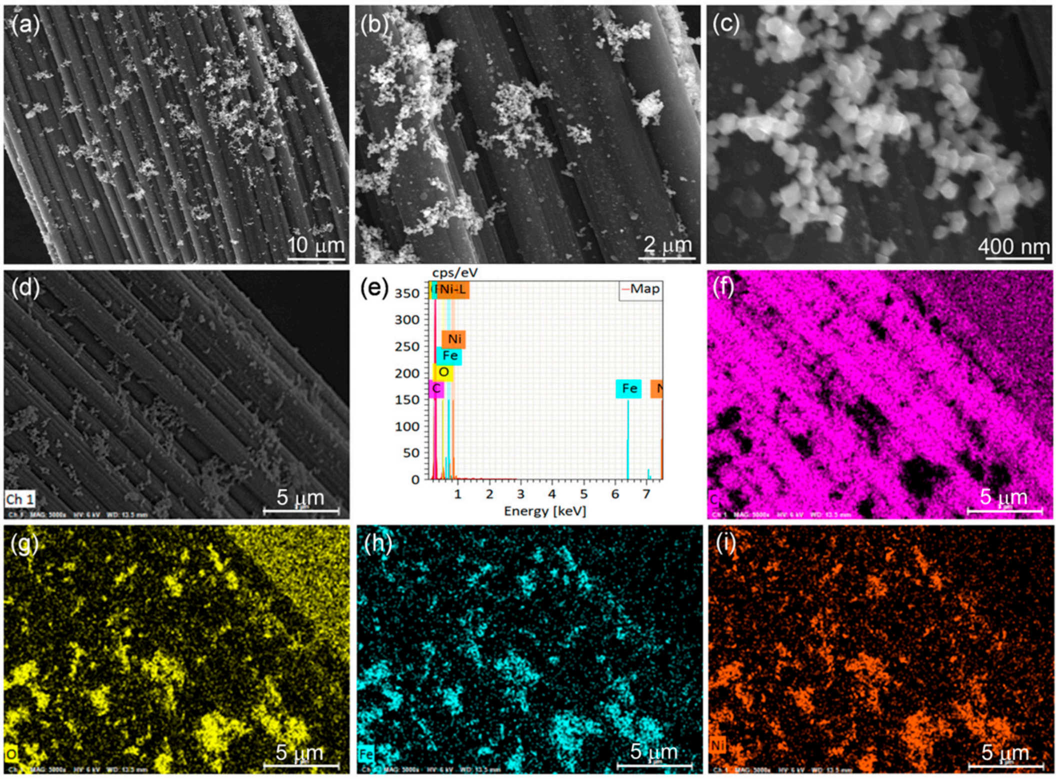

Figure S6. (**a**–**d**) SEM images, (**e**) EDX spectra, and (**f**–**i**) elemental mapping images of $NiFe_2O_4$/ carbonized bamboo fibers.

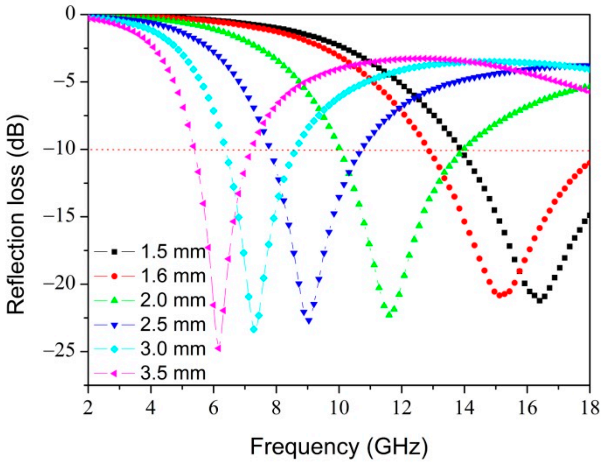

Figure S7. Frequency dependence of the simulated reflection loss for $NiFe_2O_4$/carbonized bamboo fibers at different thicknesses.
